# Level of Resilience Among Transgenders in Selected Areas of Puducherry, India: An Exploratory Research

**DOI:** 10.7759/cureus.18413

**Published:** 2021-09-30

**Authors:** Vijay K Chavada, Priyadharshini R, Jeyastri Kurushev

**Affiliations:** 1 Community Medicine, Indira Gandhi Government Medical College and Research Institute, Puducherry, IND; 2 Mental Health Nursing, Vinayaka Mission's College of Nursing Puducherry, Puducherry, IND; 3 Mental Health Nursing, Mother Theresa Post Graduate and Research Institute of Health Sciences, Puducherry, IND

**Keywords:** mental well-being, transgenders, emotional distress, health wellness, resilience and well-being

## Abstract

Background

Resilience is the process of adapting well in the face of adversity, trauma, tragedy, threats, or significant sources of stress, and it is a predictor of mental health status that specifically indicates self-esteem, perceived social support, emotion-oriented coping, and a sense of personal mastery.

The third gender known better as transgender has existed in every culture, race, class, and religion since the inception of human life has been recorded and analyzed. In spite of many advances and reforms, the current plight of transgender is far from being satisfactory. The social integration of transgender with the mainstream community is practically non-existent even today.

Aim and objective

The objective of the study is to assess the level of resilience among transgenders at selected areas in Puducherry, India, and to find out the association between the levels of resilience among transgenders with the selected demographic variables.

Methods

A descriptive cross-sectional study was conducted in the selected areas of Puducherry, India, adopting a linear snowball sampling method after consultation with the Nayaks (heads of transgenders) of the transgender groups, and 100 transgenders were enrolled who fulfilled the inclusion criteria and consented to participate in the research.

Results

About 29 (29%) transgenders were in the age group of 31-40 years, 28 (28%) were in the age group of 21-30 years, 24 (24%) were in the group of 41-50 years, and 19 (19%) were in the age group of 51 and above. In education status, 38 (38%) had secondary education, 23 (23%) had primary education, 20 (20%) had higher secondary education, 12 (12%) had graduation and above, and seven (7%) were diploma holders. Moreover, 54 (54%) were employed, and 46 (46%) were unemployed; 81 (81%) were residing in rural areas, and 19 (19%) were residing in urban areas. The study revealed that 53 (53%) of them had average resilience, 28 (28%) had the least resilience, and 19 (19%) had the highest resilience among transgenders. The minimum score was 28.0, and the maximum score was 52.0. The mean score was 42.50 with a standard deviation of 4.61. The median value was 43.0.

Conclusion

Transgenders exhibit low and average resilience that reflects poor mental health status among them. The educational status was found to be associated with the level of resilience. Proper education among transgenders would help in improving their resilience and betterment of their life.

## Introduction

Resilience is the process of adapting well in the face of adversity, trauma, tragedy, threats, or significant sources of stress such as family and relationship problems, serious health problems, or workplace and financial stressors [1]. It means "bouncing back" from difficult experiences. Resilience is a predictor of mental health status that specifically indicates self-esteem, perceived social support, emotion-oriented coping, and a sense of personal mastery [2].

It is already well established that mental illness, across the spectrum of disorders, is both a direct cause of mortality and morbidity and a significant risk factor for poorer economic, health, and social outcomes, although these adverse outcomes vary by type of disorder and socioeconomic status. However, it is now becoming clear that the presence or absence of positive mental health or “wellbeing” also influences outcomes across a wide range of domains. These include healthier lifestyles, better physical health, improved recovery, fewer limitations in daily living, higher educational attainment, greater productivity, employment and earnings, better relationships, greater social cohesion and engagement, and improved quality of life [3-5].

The third gender known better as transgender has existed in every culture, race, class, and religion since the inception of human life has been recorded and analyzed [6].

In April 2014, the Supreme Court of India declared transgender to be a “third gender” in Indian law. Transgenders are the most visible and exploited sexual minorities in India. The problems faced by transgender include discrimination in terms of education, employment, entertainment, justice; disrespect; downtrodden; child nabbing; forced to do prostitution; forced to leave the parental home; rape; verbal and physical abuse; human trafficking; and social exclusion [7]. Discrimination has prevented most transgenders from obtaining decent education, jobs, and housing, say transgender and human rights activists [8].

According to the 2011 census, the population of transgender in India is estimated to be between 50,000 and 1.2 million. There are no reliable statistics. Tamil Nadu, an Indian state, has an estimated population of 30,000 transgender people. According to the 2011 census, there are 252 transgenders in Puducherry.

Even with so many advances and reforms, the current plight of transgender is far from being satisfactory. The social integration of transgender with the mainstream community is practically non-existent even today. It is not uncommon in an everyday scenario where one usually encounters transgender begging in the metro trains, local trains, bus stands and terminuses, and other similar public places for their livelihood. This is because they are isolated and are being pushed into begging and prostitution, of which the latter makes them prone to sexually transmitted diseases (STDs) and human immunodeficiency virus diseases (HIVs). As per the WHO report on transgender, transgenders are 49 times more likely to be living with HIV than other adults of reproductive age with an estimated worldwide HIV prevalence of 19% [9].

Further, the literature has reported a high rate of suicidal tendencies among the transgender community, which ranges from 32% to 50%, and the suicide rate is about 30%; 26% of them are at high risk for major depression, and 31% and 15% are at high risk for tobacco and alcohol abuse, respectively. Hence it is evident that the transgender population is having poor mental status [10].

Moreover, a report on transgender people published by Joint United Nations Programme on HIV/AIDS (UNAIDS) in 2014 mentioned that 65%-85% of transgender people experienced verbal abuse, 25%-45% faced physical abuse, and almost 20% were sexually abused. Many of them experienced abuses from childhood to the rest of their life in a higher percentage compared to the general population [11].

Hence, there is a huge scope for research in exploring and understanding the unique mental health and psychological aspects of the transgender community in the Indian context. Till now, there are very rare studies conducted in Puducherry for assessment of the resilience among transgender. So, the current study was planned to assess the resilience among transgenders.

## Materials and methods

This study was conducted at Puducherry, India, with the following objectives: to assess the level of resilience among transgender at selected areas in Puducherry, India, and to find out the association between the level of resilience among transgender with the selected demographic variables.

For operational purposes, “Resilience” refers to the capacity to recover from difficult experiences in their life assessed through the Connor-Davidson resilience scale. Transgender refers to people who identify themselves as females though their assigned sex at birth was male [1].

Methodology

A descriptive cross-sectional study design was adopted in the selected areas of Puducherry as referred by the Nayaks (heads of transgenders) during 2020. Transgender residing in the areas of Ariyankuppam, Chinna Veerampattinam, Dubburayapet, Kurumapet, Bahour, Iyyankuttipalayam, Pitchaveeranpet, Guruvappa Nayakkan Palayam, Villianoor Manavely, Auroville, Pooranakuppam, Gopalankadai, Odiyampet, Arumparthapuram, Kanuvapet, Ilango Nagar, and Uppalam were recruited for the study. The distance of the selected areas ranged between 8 and 10 kilometers from the investigator’s institutions. The samples of this study were transgenders residing at selected areas of Puducherry those who met the inclusion criteria and those who were available at the time of data collection.

**Figure 1:**
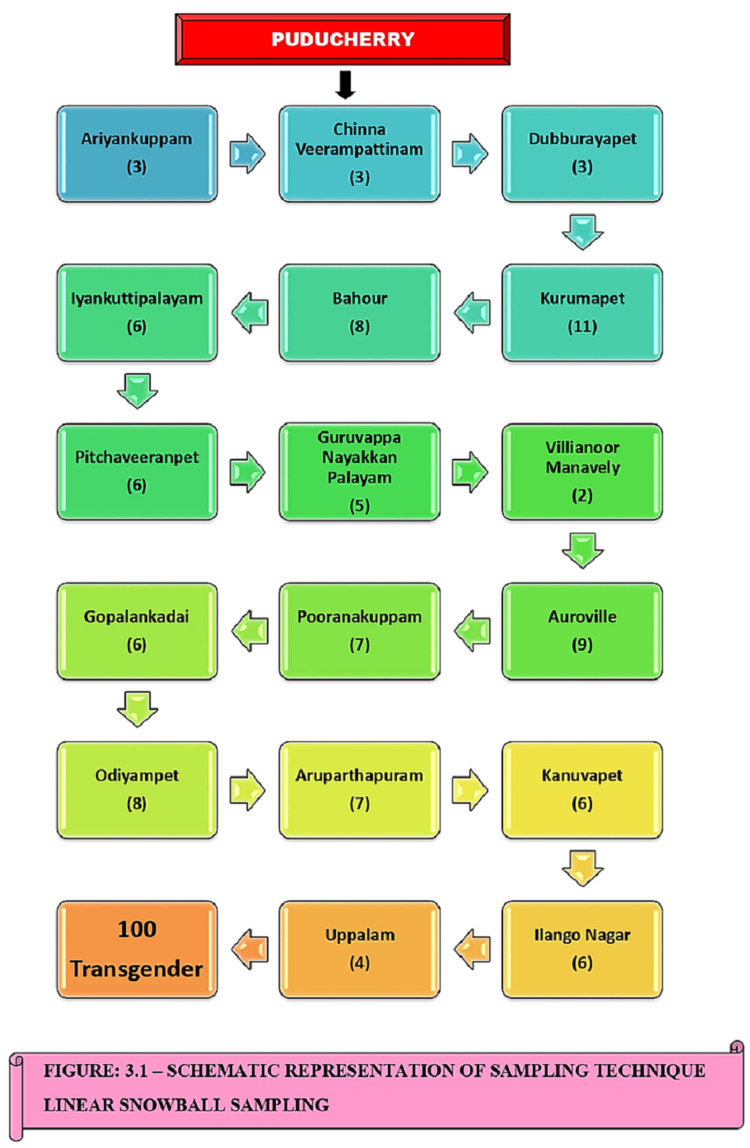
Schematic representation of linear snowball sampling technique

A hundred transgenders in the selected areas were enrolled in the study, considering the prevalence of suicidal tendency among the transgenders as 32% as reported in a study with 95% confidence level and a corresponding Z score of 1.96 at a 5% significance level with 10% nonresponse rate and using OpenEpi software version 3.2. Hence, the sample size was calculated as 100. Linear snowball sampling technique was used for the study (Figure 1). In the linear snowball sampling method, after catching the first participant (Nayak), subsequent participants were traced using the information provided by the earlier participant. Each selected sample was asked to provide a reference of only one similar subject, where the linear chain was created. Thus it was continued till the investigator reached a number of 100 samples.

Transgenders who were aged above 20 years and able to read, speak, and write in Tamil were included in the study, while transgenders who were not willing to participate, not ready to give consent, or were physically sick or terminally ill were excluded.

The data collection tool used for this study was the standardized self-administered questionnaire. After extensive literature review, internet search, and experts advice, the researcher identified the Connor-Davidson resilience scale, a standardized tool to assess resilience. The researcher obtained permission from the author Jonathan Davidson to use the tool in both English and Tamil through an agreement. The tool was divided into two parts as follows: (1) Section A - socio-demographic profile, including the age, educational status, occupation, habitat, living arrangement, religion, monthly income, number of dependents, and substance abuse; (2) Section B - Connor-Davidson resilience scale to assess the resilience among transgenders.

It consists of 25 items with a five-point Likert scale ranging from 0 to 4, and the full range is therefore from 0 to 100, with higher scores reflecting the highest resilience.

Score interpretation

Table 1 shows the interpretation of the score, and Table 2 describes the quartile. The scores were interpreted through median and quartile scores. The median describes the midpoint of the frequency distribution, and it is the point at which exactly half of the data lies below and above the central value. Quartile scores describe four groups of equal numbers taken from the observed distribution of scores. Each quartile contains 25% of the total observations.

**Table 1:** Interpretation of score

Score	Interpretation
0	Not at all true
1	Rarely true
2	Sometimes true
3	Often true
4	True nearly all of the time

**Table 2:** Description of quartiles

Quartile	Description
First quartile (Q1): the lowest 25% of numbers	Least resilient
Second and third quartiles (Q2 & Q3): between 25.1% and 75%	Average resilient
Fourth quartile (Q4): the highest 25% of numbers	Highest resilient

The validity was obtained from the experts in the field of psychiatry, psychiatric nursing, psychology, community medicine, and psychiatric social work. Opinions and suggestions given by the experts were incorporated. The reliability was established by means of the test-retest method. The obtained data were evaluated for the calculation of the correlation coefficient, and the “r” value was found to be 0.971. This indicated that the tool was highly reliable. Hence, all items appeared to be worthy of retention. Ethical clearance was obtained from the Institutional Ethical Committee of Mother Theresa Post Graduate and Research Institute of Health Sciences for conducting the study. Permission was obtained from the Nayaks. Informed consent was obtained from the study participants. The assurance was given to the study subjects that collected information will be kept confidential and anonymity will be maintained. A total of 100 transgenders were selected through the linear snowball sampling technique. The investigator introduced herself and explained the purpose of the study and obtained informed written consent from the participating transgenders. Data were collected from the transgenders with the help of the standardized self-administered questionnaire that comprised of socio-demographic data and the Connor-Davidson resilience scale to assess the level of resilience among transgenders. The subjects were requested to give appropriate responses to each question. The questionnaire was filled by the samples, and explanations were given to them whenever they had doubts. Each day the investigator collected five to six samples, and it took approximately 30 to 45 minutes to collect each sample. The same procedure was followed, and it took four weeks to complete the data collection.

The data were entered in Microsoft Excel (Microsoft Corporation, New Mexico, USA). The collected data were refined, coded, and recoded in the Statistical Package for the Social Sciences (SPSS) version 21.0 (IBM Corp., Armonk, NY). Descriptive statistics such as frequency distribution, percentage, mean, median, and standard deviation were used to describe the socio-demographic variables and assessment of the level of resilience among transgenders. For inferential statistics, a chi-squared test was used to find the association between the demographic variables and the level of resilience.

## Results

Distribution of the demographic variables of transgender

Distribution of Transgender Based on Age (n = 100)

**Figure 2:**
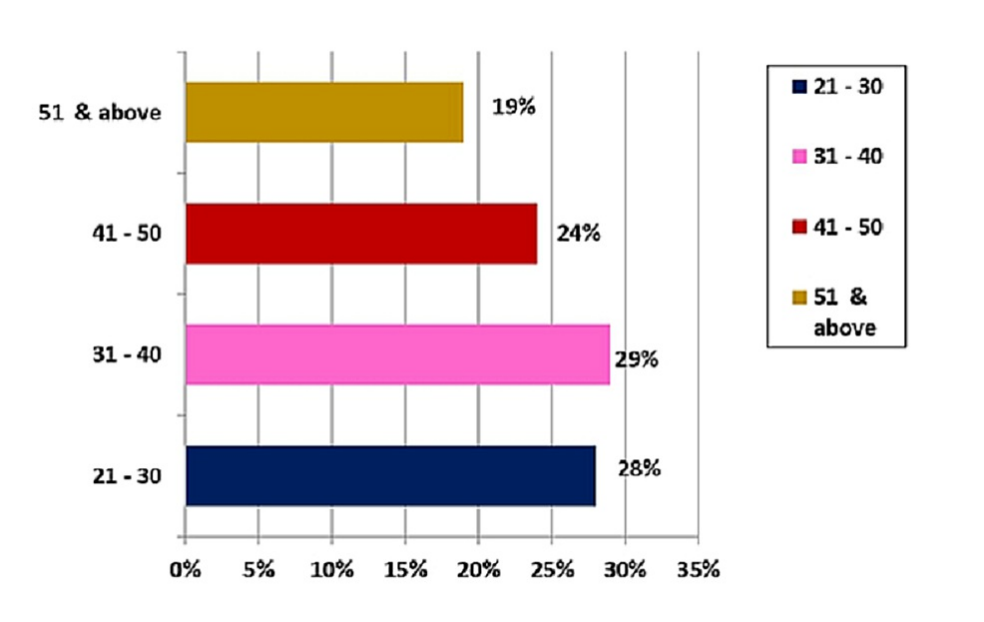
Distribution as per age group

Table 3 and Figure 2 show the frequency and percentage distribution of transgenders based on age. With regard to age (years), 29 (29%) were in the age group of 31-40, 28 (28%) were in the age group of 21-30, 24 (24%) were in the age group of 41-50 years, and 19 (19%) were in the age group of 51 and above.

**Table 3:** Distribution of participants as per age group

Age in years	N	%
21–30	28	28.0
31–40	29	29.0
4–50	24	24.0
51 and above	19	19.0

Distribution of Transgender Based on Educational Status (n = 100)

Table 4 depicts the frequency and percentage distribution of transgenders based on educational status. With respect to educational status, 38 (38%) had secondary education, 23 (23%) had primary education, 20 (20%) had higher secondary education, 12 (12%) had graduation and above, and seven (7%) were diploma holders.

**Table 4:** Distribution as per education status

Educational Status	N	%
Primary education	23	23
Secondary education	38	38
Higher secondary education	20	20
Diploma	7	7
Graduation and above	12	12

Distribution of Transgender Based on Occupation (n = 100) and Habitat (n = 100)

Considering the occupation, 54 (54%) were employed and 46 (46%) were unemployed. Regarding habitat, 81 (81%) were residing in rural areas and 19 (19%) were residing in urban areas.

Distribution of Transgender Based on Living Arrangement

With respect to the living arrangements, Table 5 shows that 72 (72%) were living with the transgender community, 13 (13%) were living with the family of origin, seven (7%) were living alone and in institutions, and only one (1%) was living in another arrangement.

**Table 5:** Distribution as per living arrangements

Living Arrangement	N	%
In the family of origin	13	13
Transgender community	72	72
Living alone	7	7
Institutionalized	7	7
Others	1	1

Distribution of Transgender Based on Religion

Regarding religion, Table 6 shows that 82 (82%) were Hindus, 11 (11%) were Christians, six (6%) were Muslims, and only one (1%) belonged to another religion.

**Table 6:** Distribution as per religion

Religion	N	%
Hindu	82	82
Christian	11	11
Muslim	6	6
Others	1	1

Distribution of Transgender Based on Monthly Income

As per Table 7, considering the monthly income (in rupees), 44 (44%) had a monthly income of 5001-10000, 29 (29%) had a monthly income of 10,001-15,000, 14 (14%) had a monthly income of 5,000, 11 (11%) had a monthly income of 15,001-20,000, and only two (2%) had a monthly income of >20,001.

**Table 7:** Distribution as per monthly income

Monthly Income (Indian Rupees Per Month)	N	%
5,000	14	14
5,001–10,000	44	44
10,001–15,000	29	29
15,001–20,000	11	11
>20,001	2	2

Distribution of Transgender Based on Number of Dependents (n = 100)

With regard to the number of dependents, Table 8 indicates 38 (38%) of them had no dependent, 30 (30%) had one dependent, 17 (17%) had two dependents, 11 (11%) had three dependents, and four (4%) had more than three dependents.

**Table 8:** Distribution as per the number of dependents

Number of Dependents	N	%
Nil	38	38
1	30	30
2	17	17
3	11	11
>3	4	4

Distribution of Transgender Based on Substance Use (n = 100)

Table 9 shows that 35 (35%) participants had no substance abuse, 26 (26%) had the habit of consuming alcohol, 14 (14%) had used both alcohol and tobacco, 13 (13%) had used tobacco, and 12 (12%) had used other substances.

**Table 9:** Distribution as per substance use

Substance Use	N	%
Nil	35	35
Alcohol	26	26
Tobacco	13	13
Alcohol and tobacco	14	14
Others	12	12

Assessment of the level of resilience among transgenders

Descriptive Statistics of Resilience Score (n = 100)

Table 10 shows the calculation of mean, median, standard deviation, minimum and maximum scores of resilience among transgenders. This table shows that the minimum resilience score was 28.0, and the maximum resilience score was 52.0. The mean resilience score was 42.50 with a standard deviation of 4.61. The median value was 43.0, and it is the midpoint of the frequency distribution based on which the four groups of quartile scores were determined.

**Table 10:** Descriptive statistics of resilience score

Resilience	Parameters
Minimum	28.0
Maximum	52.0
Mean	42.51
Median	43.0
Standard deviation	4.61

Distribution of the Level of Resilience Among Transgenders (n = 100)

**Figure 3:**
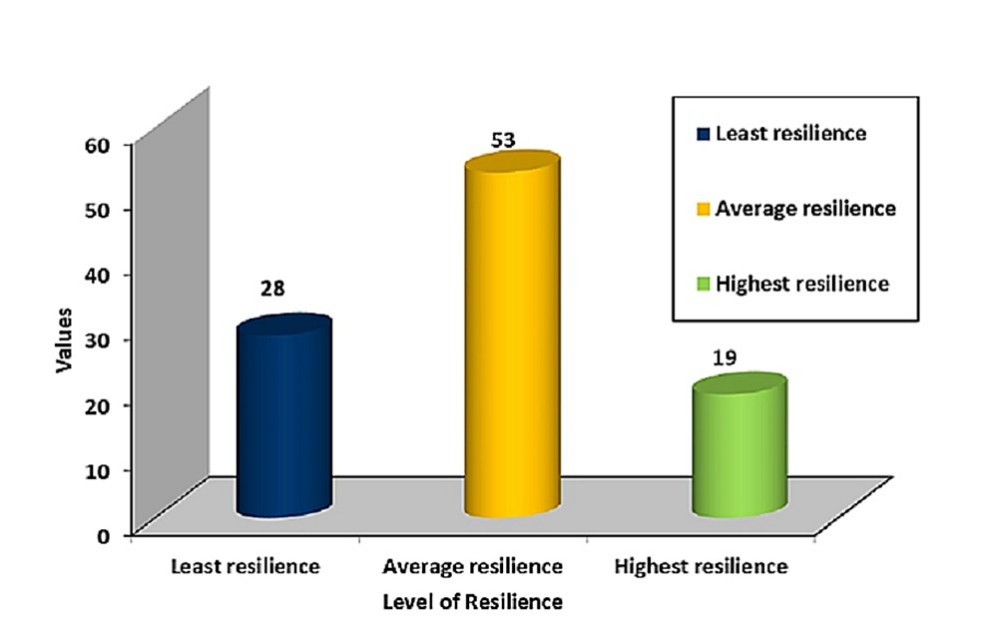
Distribution as per the level of resilience

Table 11 and Figure 3 depict the frequency and percentage distribution of the level of resilience among transgenders. It is evident from the above table that 53 (53%) of the participants had average resilience, 28 (28%) had the least resilience, and 19 (19%) had the highest resilience among transgenders.

**Table 11:** Distribution as per the level of resilience

Level of Resilience	N	%
Least resilience (1^st^ quartile)	28	28.0
Average resilience (2^nd ^and 3^rd^ quartiles)	53	53.0
Highest resilience (4^th^ quartile)	19	19.0

Association of the Level of Resilience Among Transgenders With Their Selected Demographic Variables (n = 100)

Tables 12, 13 show that the demographic variable educational status had shown statistically significant association with the level of resilience among transgenders with chi-square values of (c^2^ = 17.218, p = 0.028) at p < 0.05 level, and the other demographic variables had not shown statistically significant association with the level of resilience among transgenders.

**Table 12:** Association of age, educational level, occupation, habitat, and religion with the level of resilience among transgenders c^2^, Chi-square values; N.S., non-significance.

Demographic Variables	Least		Moderate		High		Sig.
	N	%	N	%	N	%	
Age (years)							c^2 ^= 8.623, p = 0.196 N.S.
21–30	12	12	22	22	7	7	
31–40	13	13	18	18	4	4	
41–50	3	3	9	9	7	7	
51 and above	0	0	4	4	1	1	
Educational status							c^2 ^= 17.218, p = 0.028 S*
Primary education	6	6	11	11	6	6	
Secondary education	15	15	16	16	7	7	
Higher secondary education	3	3	16	16	1	1	
Diploma	1	1	2	2	4	4	
Graduation and above	3	3	8	8	1	1	
Occupation							c^2 ^= 3.455, p = 0.178 N.S.
Employed	18	18	24	24	12	12	
Unemployed	10	10	29	29	7	7	
Habitat							c^2 ^= 0.235, p = 0.889 N.S.
Urban	6	6	10	10	3	3	
Rural	22	22	43	43	16	16	
Living arrangement							c^2 ^= 6.964, p = 0.541 N.S.
In family of origin	3	30	7	7	3	3	
Transgender community	23	23	37	37	12	12	
Living alone	1	1	5	5	1	1	
Institutionalized	1	1	4	4	2	2	
Others	0	0	0	0	1	1	
Religion							c^2 ^= 10.375, p = 0.110 N.S.
Hindu	24	24	46	46	12	12	
Christian	2	2	4	4	5	5	
Muslim	2	2	3	3	1	1	
Others	0	0	0	0	1	1	

**Table 13:** Association of monthly income, number of dependents, and substance use with the level of resilience among transgenders c^2^, Chi-square values; N.S., non-significance.

Demographic Variables	Least		Moderate		High		Sig.
	N	%	N	%	N	%	
Monthly income							c^2 ^= 8.823, p = 0.357 N.S.
5,000	2	2	8	8	4	4	
5,001–10,000	12	12	25	25	7	7	
10,001–15,000	9	9	13	13	7	7	
15,001–20,000	3	3	7	7	1	1	
>20,001	2	2	0	0	0	0	
Number of dependents							c^2 ^= 4.440, p = 0.815 N.S.
Nil	11	11	19	19	8	8	
1	8	8	18	18	4	4	
2	3	3	9	9	5	5	
3	4	4	5	5	2	2	
>3	2	2	2	2	0	0	
Substance use							c^2 ^= 5.915, p = 0.657 N.S.
Nil	12	12	18	18	5	5	
Alcohol	7	7	13	13	6	6	
Tobacco	2	2	10	10	1	1	
Alcohol and tobacco	4	4	7	7	3	3	
Others	3	3	5	5	4	4	

## Discussion

Distribution of the selected demographic variables of the sample

With regard to age, more than half of the total transgenders, i.e., 57 (57%) were in the age group of 21-40 years, while another one-fourth, i.e., 28 (28%) were in the age group of 41-50 years, and 19 (19%) were in the age group of 51 and above. Two-thirds of the transgenders, i.e., 61 (61%) were educated up to either primary or secondary level of education, 46 (46%) were unemployed, and 81 (81%) were residing in rural areas. A total of 72(72%) participants were living with the transgender community, and only 13 (13%) were living with the family.

Considering the monthly income, more than half, i.e., 58 (58%) have had an income of below 15,000 per month, while two-thirds of the transgenders have had dependents ranging from at least one to more than three. Moreover, 65 (65%) of the participants have had substance abuse, either of tobacco or alcohol.

The objective of the study was to assess the level of resilience among transgenders. The level of resilience among transgenders has been assessed through the Connor-Davidson resilience scale.

The present study revealed that 53 (53%) of the participants had average resilience, 28 (28%) had the least resilience, and 19 (19%) had the highest resilience among the transgenders. The minimum score was 28.0, and the maximum score was 52.0. The mean score was 42.50 with a standard deviation of 4.61. The median value was 43.0.

The present study results were supported by a study [12] conducted, which assessed the mental resilience of transgenders. A convenient sampling technique was used. The results showed that among the 65 transgenders, about 32 had the least resilience, 28 had moderate resilience, and only five had high resilience. The researcher observed that the minimum score was 16, and the maximum score was 56. The median value was 45.0.

The second objective of the study is to associate the level of resilience with socio-demographic variables such as age, educational status, occupation, habitat, living arrangement, religion, monthly income, number of dependents, and substance abuse.

The demographic variable educational status had shown statistically significant association with the level of resilience with chi-square value of (c^2^ = 17.218, p = 0.028) at p < 0.05 level, and the other demographic variables had not shown statistically significant association with the level of resilience among transgenders.

This study’s findings were found to be consistent with the study conducted by Pandya et al. [13] to assess the resilience of transgenders, an Indian perspective. Sixty transgender persons were recruited for the study through the consecutive sampling method. The Connor-Davidson resilience scale was administered. Respondents have scored low (59.30 ± 15.02912) on the resilience scale, and this score is lower than any other population scored on this scale across the world. This suggests a poor resilience status of the respondents. Residing at the family of origin or in the mainstream, having higher education status, and being employed are the factors associated with better resilience among transgenders.

Transgenders experience considerable emotional distress and have a poor social quality of life. The cause of emotional distress and poorer quality of life can be manifold including stigma, discrimination, isolation, lack of educational and employment opportunities, and victimization.

## Conclusions

The study findings indicated that transgenders exhibit low and average resilience, which reflects poor mental health status among them. The results revealed that educational status was found to be associated with the level of resilience. Proper education among transgenders will help in improving their resilience and betterment of their life. Moreover, the researcher suggests that in especially those with least or minimal resilience, protective factors like acceptance, social support, counseling, employment, proper healthcare services, and social inclusion may improve and progress transgender’s resilience. Therefore, it can be concluded that the present study set statistics about the resilience among transgenders. Mental health professionals have a great responsibility to respond to this and initiate appropriate interventions such as resilience-building programs, psychoeducation, counseling, etc.
